# Methyl 3-(4-methoxy­benzo­yl)propionate

**DOI:** 10.1107/S1600536808037720

**Published:** 2008-11-20

**Authors:** Sajid Ali, Ghulam Qadeer, Nasim Hasan Rama, Wai-Yeung Wong

**Affiliations:** a Department of Chemistry, Quaid-i-Azam University, Islamabad 45320, Pakistan; b Department of Chemistry, Hong Kong Baptist University, Waterloo Road, Kowloon Tong, Hong Kong

## Abstract

The asymmetric unit of the title compound, C_12_H_14_O_3_, contains two independent mol­ecules, in which the benzene rings are oriented at a dihedral angle of 72.08 (3)°. In the crystal structure, inter­molecular C—H⋯O hydrogen bonds link the mol­ecules into centrosymmetric dimers. There are also C—H⋯π contacts between aromatic CH groups and the benzene rings.

## Related literature

For general background, see: Hashem *et al.* (2007 ▶); Husain *et al.*(2005 ▶). For a related structure, see: Ali *et al.* (2008 ▶). For bond-length data, see: Allen *et al.* (1987 ▶).
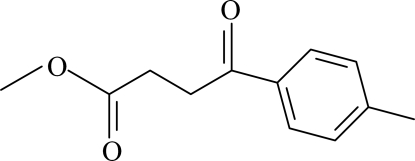


## Experimental

### 

#### Crystal data

C_12_H_14_O_3_
*M* *_r_* = 206.23
Monoclinic, 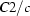

*a* = 34.762 (4) Å
*b* = 5.2861 (7) Å
*c* = 27.752 (3) Å
β = 117.182 (2)°
*V* = 4536.5 (9) Å^3^
*Z* = 16
Mo *K*α radiation
μ = 0.09 mm^−1^
*T* = 294 (2) K
0.28 × 0.26 × 0.23 mm

#### Data collection

Bruker SMART CCD area-detector diffractometer
Absorption correction: multi-scan (*SADABS*; Bruker, 1998 ▶) *T*_min_ = 0.798, *T*_max_ = 0.980
13099 measured reflections
5456 independent reflections
3364 reflections with *I* > 2σ(*I*)
*R*_int_ = 0.025

#### Refinement

*R*[*F* ^2^ > 2σ(*F* ^2^)] = 0.049
*wR*(*F* ^2^) = 0.174
*S* = 1.01
5456 reflections
272 parameters
H-atom parameters constrained
Δρ_max_ = 0.25 e Å^−3^
Δρ_min_ = −0.18 e Å^−3^

### 

Data collection: *SMART* (Bruker, 1998 ▶); cell refinement: *SAINT* (Bruker, 1999 ▶); data reduction: *SAINT*; program(s) used to solve structure: *SHELXS97* (Sheldrick, 2008 ▶); program(s) used to refine structure: *SHELXL97* (Sheldrick, 2008 ▶); molecular graphics: *PLATON* (Spek, 2003 ▶); software used to prepare material for publication: *SHELXL97*.

## Supplementary Material

Crystal structure: contains datablocks I, global. DOI: 10.1107/S1600536808037720/hk2571sup1.cif

Structure factors: contains datablocks I. DOI: 10.1107/S1600536808037720/hk2571Isup2.hkl

Additional supplementary materials:  crystallographic information; 3D view; checkCIF report

## Figures and Tables

**Table 1:** Hydrogen-bond geometry (Å, °)

*D*—H⋯*A*	*D*—H	H⋯*A*	*D*⋯*A*	*D*—H⋯*A*
C24—H24*A*⋯O1^i^	0.96	2.53	3.467 (3)	164
C4—H4*A*⋯*Cg*1^ii^	0.93	3.17	3.858 (4)	133
C6—H6*A*⋯*Cg*2^iii^	0.93	3.26	4.051 (3)	144
C18—H18*A*⋯*Cg*1	0.93	3.20	3.940 (3)	138

Symmetry codes: (i) 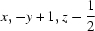; (ii) 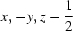; (iii) 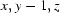. *Cg*1 and *Cg*2 are the centroids of the C2–C7 and C14–C19 rings.

**Fig. 1.**
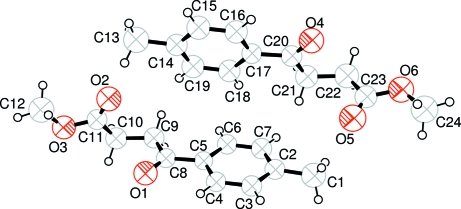
The molecular structure of the title molecule, with the atom-numbering scheme.

**Fig. 2.**
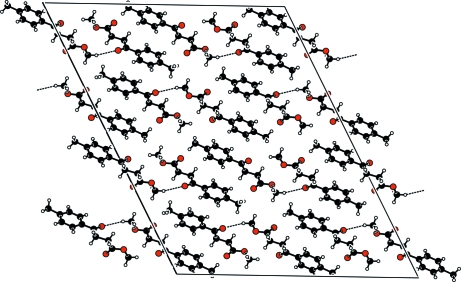
A partial packing diagram. Hydrogen bonds are shown as dashed lines.

## supplementary crystallographic information

### Comment

Benzoyl propionic acids and esters are important intermediates in heterocyclic chemistry and have been used for the synthesis of various biologically active five-membered heterocyles such as butenolides, pyrrolones (Husain *et al.*,  2005), oxadiazoles and triazoles (Hashem *et al.*,  2007). In view of the versatility of these compounds, we synthesized the title compound and reported herein its crystal structure.

The asymmetric unit of the title compound contains two crystallographically independent molecules of similar geometry (Fig.1). The bond lengths (Allen *et al.*,  1987) and angles are within normal ranges and are comparable with the corresponding values in 3-(4-methoxybenzoyl)propionic acid (Ali *et al.*, 2008). Rings A (C2-C7) and B (C14-C19) are, of course, planar and they are oriented at a dihedral angle of 72.08 (3)°.

In the crystal structure, intermolecular C-H···O hydrogen bonds (Table 1) link the molecules into centrosymmetric dimers (Fig. 2), in which they may be effective in the stabilization of the structure. There also exist C—H···π contacts (Table 1) between the aromatic CH groups and the benzene rings.

### Experimental

For the preparation of the title compound, the mixture of 3-(4-methoxybenzoyl) propionic acid (2.08 g, 10 mmol) and absolute methanol (50 ml) in the presence of a few drops of suphuric acid was refluxed for 5 h. The excess of solvent was removed by distillation. The solid residue for filltered off, washed with water and recystallized from ethanol (30%) to give the title compound. Crystals suitable for X-ray analysis were obtained by slow evaporation of an ethanol solution at room temperature (yield; 83%, m.p. 308-309 K).

### Refinement

H atoms were positioned geometrically, with C-H = 0.93, 0.97 and 0.96 Å for aromatic, methylene and methyl H, respectively, and constrained to ride on their parent atoms with U_iso_(H) = xU_eq_(C), where x = 1.5 for methyl H and x = 1.2 for all other H atoms.

### Crystal data

**Table:**

C_12_H_14_O_3_	*F*_000_ = 1760
*M**_r_* = 206.23	*D*_x_ = 1.208 Mg m^−^^3^
Monoclinic, *C*2/*c*	Melting point: 308(1) K
Hall symbol: -C 2yc	Mo *K*α radiation λ = 0.71073 Å
*a* = 34.762 (4) Å	Cell parameters from 2335 reflections
*b* = 5.2861 (7) Å	θ = 5.3–18.6º
*c* = 27.752 (3) Å	µ = 0.09 mm^−^^1^
β = 117.182 (2)º	*T* = 294 (2) K
*V* = 4536.5 (9) Å^3^	Block, colorless
*Z* = 16	0.28 × 0.26 × 0.23 mm

### Data collection

**Table:**

Bruker SMART CCD area-detector diffractometer	5456 independent reflections
Radiation source: fine-focus sealed tube	3364 reflections with *I* > 2σ(*I*)
Monochromator: graphite	*R*_int_ = 0.025
*T* = 294(2) K	θ_max_ = 28.3º
ω and φ scans	θ_min_ = 2.4º
Absorption correction: multi-scan(SADABS; Bruker, 1998)	*h* = −37→45
*T*_min_ = 0.798, *T*_max_ = 0.980	*k* = −6→6
13099 measured reflections	*l* = −37→27

### Refinement

**Table:**

Refinement on *F*^2^	Hydrogen site location: inferred from neighbouring sites
Least-squares matrix: full	H-atom parameters constrained
*R*[*F*^2^ > 2σ(*F*^2^)] = 0.049	*w* = 1/[σ^2^(*F*_o_^2^) + (0.0886*P*)^2^ + 0.985*P*] where *P* = (*F*_o_^2^ + 2*F*_c_^2^)/3
*wR*(*F*^2^) = 0.174	(Δ/σ)_max_ < 0.001
*S* = 1.01	Δρ_max_ = 0.25 e Å^−^^3^
5456 reflections	Δρ_min_ = −0.18 e Å^−^^3^
272 parameters	Extinction correction: SHELXL97 (Sheldrick, 2008), Fc^*^=kFc[1+0.001xFc^2^λ^3^/sin(2θ)]^-1/4^
Primary atom site location: structure-invariant direct methods	Extinction coefficient: 0.0021 (4)
Secondary atom site location: difference Fourier map	

### Special details

**Table:**

Geometry. All e.s.d.'s (except the e.s.d. in the dihedral angle between two l.s. planes) are estimated using the full covariance matrix. The cell e.s.d.'s are taken into account individually in the estimation of e.s.d.'s in distances, angles and torsion angles; correlations between e.s.d.'s in cell parameters are only used when they are defined by crystal symmetry. An approximate (isotropic) treatment of cell e.s.d.'s is used for estimating e.s.d.'s involving l.s. planes.
Refinement. Refinement of *F*^2^ against ALL reflections. The weighted *R*-factor *wR* and goodness of fit *S* are based on *F*^2^, conventional *R*-factors *R* are based on *F*, with *F* set to zero for negative *F*^2^. The threshold expression of *F*^2^ > σ(*F*^2^) is used only for calculating *R*-factors(gt) *etc*. and is not relevant to the choice of reflections for refinement. *R*-factors based on *F*^2^ are statistically about twice as large as those based on *F*, and *R*- factors based on ALL data will be even larger.

### Fractional atomic coordinates and isotropic or equivalent isotropic displacement parameters (Å^2^)

**Table:**

	*x*	*y*	*z*	*U*_iso_*/*U*_eq_	
O1	0.17237 (4)	0.1342 (3)	0.28466 (5)	0.0856 (4)	
O2	0.07042 (4)	0.0750 (3)	0.24528 (5)	0.0908 (4)	
O3	0.08802 (4)	−0.0612 (3)	0.32828 (5)	0.0849 (4)	
O4	0.07570 (4)	0.7301 (3)	−0.03309 (5)	0.0848 (4)	
O5	0.17800 (5)	0.7032 (3)	0.00217 (5)	0.0987 (5)	
O6	0.16293 (5)	0.6067 (3)	−0.08206 (5)	0.0923 (5)	
C1	0.23566 (7)	0.1919 (5)	0.09882 (8)	0.0915 (6)	
H1A	0.2259	0.0684	0.0703	0.137*	
H1B	0.2665	0.1819	0.1196	0.137*	
H1C	0.2277	0.3579	0.0834	0.137*	
C2	0.21493 (5)	0.1408 (3)	0.13526 (6)	0.0633 (4)	
C3	0.22438 (5)	0.2949 (3)	0.17972 (6)	0.0633 (4)	
H3A	0.2427	0.4326	0.1860	0.076*	
C4	0.20724 (5)	0.2485 (3)	0.21467 (6)	0.0592 (4)	
H4A	0.2144	0.3537	0.2444	0.071*	
C5	0.17922 (4)	0.0452 (3)	0.20601 (6)	0.0527 (3)	
C6	0.16911 (5)	−0.1061 (3)	0.16102 (6)	0.0622 (4)	
H6A	0.1502	−0.2413	0.1541	0.075*	
C7	0.18676 (6)	−0.0588 (3)	0.12648 (6)	0.0683 (4)	
H7A	0.1796	−0.1633	0.0966	0.082*	
C8	0.16253 (5)	−0.0046 (3)	0.24590 (6)	0.0584 (4)	
C9	0.13441 (6)	−0.2324 (3)	0.23814 (7)	0.0695 (4)	
H9A	0.1499	−0.3823	0.2367	0.083*	
H9B	0.1087	−0.2176	0.2036	0.083*	
C10	0.12090 (6)	−0.2663 (4)	0.28236 (8)	0.0749 (5)	
H10A	0.1070	−0.4296	0.2779	0.090*	
H10B	0.1465	−0.2659	0.3172	0.090*	
C11	0.09074 (5)	−0.0647 (3)	0.28213 (7)	0.0645 (4)	
C12	0.05918 (7)	0.1240 (5)	0.33221 (9)	0.0975 (7)	
H12A	0.0597	0.1109	0.3670	0.146*	
H12B	0.0303	0.0942	0.3043	0.146*	
H12C	0.0683	0.2904	0.3280	0.146*	
C13	0.01362 (6)	0.5636 (5)	0.15220 (8)	0.0921 (6)	
H13A	−0.0064	0.7016	0.1435	0.138*	
H13B	0.0360	0.5820	0.1887	0.138*	
H13C	−0.0013	0.4068	0.1488	0.138*	
C14	0.03334 (5)	0.5641 (3)	0.11394 (7)	0.0663 (4)	
C15	0.02252 (6)	0.7474 (4)	0.07450 (7)	0.0760 (5)	
H15A	0.0030	0.8735	0.0719	0.091*	
C16	0.04011 (5)	0.7474 (3)	0.03875 (7)	0.0717 (5)	
H16A	0.0321	0.8726	0.0124	0.086*	
C17	0.06942 (5)	0.5640 (3)	0.04171 (6)	0.0572 (4)	
C18	0.08110 (6)	0.3822 (3)	0.08218 (7)	0.0708 (5)	
H18A	0.1011	0.2582	0.0855	0.085*	
C19	0.06326 (6)	0.3842 (4)	0.11747 (7)	0.0750 (5)	
H19A	0.0716	0.2612	0.1443	0.090*	
C20	0.08610 (5)	0.5643 (3)	0.00094 (6)	0.0631 (4)	
C21	0.11550 (6)	0.3537 (4)	0.00230 (8)	0.0770 (5)	
H21A	0.1011	0.1937	−0.0001	0.092*	
H21B	0.1413	0.3577	0.0369	0.092*	
C22	0.12873 (7)	0.3661 (4)	−0.04260 (9)	0.0868 (6)	
H22A	0.1424	0.2072	−0.0435	0.104*	
H22B	0.1029	0.3841	−0.0769	0.104*	
C23	0.15891 (6)	0.5766 (4)	−0.03702 (7)	0.0709 (5)	
C24	0.19204 (7)	0.8028 (5)	−0.08167 (9)	0.1000 (7)	
H24A	0.1923	0.8066	−0.1161	0.150*	
H24B	0.2207	0.7686	−0.0536	0.150*	
H24C	0.1825	0.9635	−0.0751	0.150*	

### Atomic displacement parameters (Å^2^)

**Table:**

	*U*^11^	*U*^22^	*U*^33^	*U*^12^	*U*^13^	*U*^23^
O1	0.1036 (9)	0.0928 (10)	0.0726 (8)	−0.0206 (8)	0.0509 (7)	−0.0246 (7)
O2	0.1021 (9)	0.1002 (11)	0.0765 (8)	0.0355 (8)	0.0462 (7)	0.0253 (7)
O3	0.0976 (9)	0.0987 (10)	0.0740 (8)	0.0180 (8)	0.0528 (7)	0.0146 (7)
O4	0.1052 (9)	0.0775 (9)	0.0786 (8)	0.0147 (7)	0.0478 (7)	0.0208 (7)
O5	0.1186 (11)	0.1140 (12)	0.0730 (8)	−0.0327 (9)	0.0522 (8)	−0.0226 (8)
O6	0.1053 (10)	0.1139 (12)	0.0700 (8)	−0.0022 (9)	0.0507 (7)	−0.0093 (8)
C1	0.0910 (13)	0.1117 (17)	0.0855 (13)	0.0006 (12)	0.0522 (11)	0.0034 (12)
C2	0.0589 (9)	0.0697 (10)	0.0598 (9)	0.0106 (7)	0.0259 (7)	0.0069 (8)
C3	0.0572 (8)	0.0606 (9)	0.0672 (9)	−0.0006 (7)	0.0242 (7)	0.0019 (8)
C4	0.0594 (8)	0.0547 (9)	0.0586 (8)	0.0005 (7)	0.0225 (7)	−0.0069 (7)
C5	0.0541 (7)	0.0487 (8)	0.0509 (7)	0.0080 (6)	0.0201 (6)	0.0019 (6)
C6	0.0713 (9)	0.0529 (9)	0.0595 (9)	−0.0035 (7)	0.0273 (7)	−0.0051 (7)
C7	0.0822 (10)	0.0653 (10)	0.0573 (9)	−0.0004 (8)	0.0316 (8)	−0.0091 (7)
C8	0.0605 (8)	0.0548 (9)	0.0576 (8)	0.0072 (7)	0.0249 (7)	−0.0006 (7)
C9	0.0812 (11)	0.0573 (10)	0.0812 (11)	0.0029 (8)	0.0466 (9)	−0.0008 (8)
C10	0.0902 (12)	0.0629 (10)	0.0844 (12)	0.0094 (9)	0.0510 (10)	0.0145 (9)
C11	0.0683 (9)	0.0653 (10)	0.0650 (9)	−0.0016 (8)	0.0348 (8)	0.0059 (8)
C12	0.1033 (15)	0.1157 (18)	0.0954 (15)	0.0148 (14)	0.0644 (13)	−0.0035 (13)
C13	0.0812 (12)	0.1145 (18)	0.0849 (13)	−0.0088 (12)	0.0416 (10)	0.0061 (12)
C14	0.0616 (9)	0.0695 (11)	0.0609 (9)	−0.0118 (8)	0.0219 (7)	−0.0007 (8)
C15	0.0741 (10)	0.0726 (12)	0.0813 (12)	0.0137 (9)	0.0355 (9)	0.0095 (9)
C16	0.0768 (10)	0.0646 (11)	0.0719 (10)	0.0135 (8)	0.0324 (9)	0.0178 (8)
C17	0.0585 (8)	0.0463 (8)	0.0573 (8)	−0.0045 (6)	0.0182 (7)	0.0001 (6)
C18	0.0784 (10)	0.0547 (9)	0.0737 (11)	0.0096 (8)	0.0301 (9)	0.0094 (8)
C19	0.0878 (12)	0.0652 (11)	0.0684 (10)	0.0022 (9)	0.0324 (9)	0.0167 (8)
C20	0.0691 (9)	0.0529 (9)	0.0616 (9)	−0.0059 (7)	0.0250 (7)	−0.0015 (7)
C21	0.0949 (12)	0.0555 (10)	0.0905 (13)	0.0031 (9)	0.0510 (11)	0.0012 (9)
C22	0.1103 (14)	0.0699 (12)	0.0914 (13)	−0.0015 (11)	0.0560 (12)	−0.0178 (10)
C23	0.0805 (11)	0.0753 (12)	0.0622 (10)	0.0100 (9)	0.0372 (9)	−0.0041 (8)
C24	0.1055 (15)	0.1219 (19)	0.0944 (15)	0.0072 (14)	0.0645 (13)	0.0115 (14)

### Geometric parameters (Å, °)

**Table:**

C1—C2	1.510 (2)	C13—C14	1.504 (2)
C1—H1A	0.9600	C13—H13A	0.9600
C1—H1B	0.9600	C13—H13B	0.9600
C1—H1C	0.9600	C13—H13C	0.9600
C2—C7	1.384 (2)	C14—C15	1.379 (2)
C2—C3	1.387 (2)	C14—C19	1.379 (2)
C3—C4	1.373 (2)	C15—C16	1.383 (2)
C3—H3A	0.9300	C15—H15A	0.9300
C4—C5	1.396 (2)	C16—C17	1.382 (2)
C4—H4A	0.9300	C16—H16A	0.9300
C5—C6	1.386 (2)	C17—C18	1.390 (2)
C5—C8	1.489 (2)	C17—C20	1.489 (2)
C6—C7	1.377 (2)	C18—C19	1.378 (2)
C6—H6A	0.9300	C18—H18A	0.9300
C7—H7A	0.9300	C19—H19A	0.9300
C8—O1	1.2151 (19)	C20—O4	1.2159 (19)
C8—C9	1.503 (2)	C20—C21	1.500 (2)
C9—C10	1.511 (2)	C21—C22	1.514 (3)
C9—H9A	0.9700	C21—H21A	0.9700
C9—H9B	0.9700	C21—H21B	0.9700
C10—C11	1.493 (2)	C22—C23	1.488 (3)
C10—H10A	0.9700	C22—H22A	0.9700
C10—H10B	0.9700	C22—H22B	0.9700
C11—O2	1.1955 (19)	C23—O5	1.188 (2)
C11—O3	1.3268 (19)	C23—O6	1.329 (2)
C12—O3	1.440 (2)	C24—O6	1.445 (3)
C12—H12A	0.9600	C24—H24A	0.9600
C12—H12B	0.9600	C24—H24B	0.9600
C12—H12C	0.9600	C24—H24C	0.9600
			
C2—C1—H1A	109.5	C14—C13—H13B	109.5
C2—C1—H1B	109.5	H13A—C13—H13B	109.5
H1A—C1—H1B	109.5	C14—C13—H13C	109.5
C2—C1—H1C	109.5	H13A—C13—H13C	109.5
H1A—C1—H1C	109.5	H13B—C13—H13C	109.5
H1B—C1—H1C	109.5	C15—C14—C19	117.67 (16)
C7—C2—C3	117.74 (15)	C15—C14—C13	120.96 (17)
C7—C2—C1	122.31 (17)	C19—C14—C13	121.37 (17)
C3—C2—C1	119.94 (17)	C14—C15—C16	121.32 (17)
C4—C3—C2	121.40 (15)	C14—C15—H15A	119.3
C4—C3—H3A	119.3	C16—C15—H15A	119.3
C2—C3—H3A	119.3	C17—C16—C15	120.85 (16)
C3—C4—C5	120.66 (14)	C17—C16—H16A	119.6
C3—C4—H4A	119.7	C15—C16—H16A	119.6
C5—C4—H4A	119.7	C16—C17—C18	117.93 (15)
C6—C5—C4	117.99 (14)	C16—C17—C20	119.12 (14)
C6—C5—C8	122.87 (14)	C18—C17—C20	122.93 (15)
C4—C5—C8	119.11 (13)	C19—C18—C17	120.59 (16)
C7—C6—C5	120.80 (15)	C19—C18—H18A	119.7
C7—C6—H6A	119.6	C17—C18—H18A	119.7
C5—C6—H6A	119.6	C18—C19—C14	121.61 (16)
C6—C7—C2	121.38 (15)	C18—C19—H19A	119.2
C6—C7—H7A	119.3	C14—C19—H19A	119.2
C2—C7—H7A	119.3	O4—C20—C17	120.69 (15)
O1—C8—C5	120.22 (15)	O4—C20—C21	120.79 (16)
O1—C8—C9	120.82 (15)	C17—C20—C21	118.51 (14)
C5—C8—C9	118.94 (13)	C20—C21—C22	114.01 (16)
C8—C9—C10	113.66 (15)	C20—C21—H21A	108.7
C8—C9—H9A	108.8	C22—C21—H21A	108.7
C10—C9—H9A	108.8	C20—C21—H21B	108.7
C8—C9—H9B	108.8	C22—C21—H21B	108.7
C10—C9—H9B	108.8	H21A—C21—H21B	107.6
H9A—C9—H9B	107.7	C23—C22—C21	114.28 (16)
C11—C10—C9	112.96 (14)	C23—C22—H22A	108.7
C11—C10—H10A	109.0	C21—C22—H22A	108.7
C9—C10—H10A	109.0	C23—C22—H22B	108.7
C11—C10—H10B	109.0	C21—C22—H22B	108.7
C9—C10—H10B	109.0	H22A—C22—H22B	107.6
H10A—C10—H10B	107.8	O5—C23—O6	122.60 (19)
O2—C11—O3	122.84 (16)	O5—C23—C22	126.08 (17)
O2—C11—C10	125.71 (16)	O6—C23—C22	111.31 (16)
O3—C11—C10	111.44 (14)	O6—C24—H24A	109.5
O3—C12—H12A	109.5	O6—C24—H24B	109.5
O3—C12—H12B	109.5	H24A—C24—H24B	109.5
H12A—C12—H12B	109.5	O6—C24—H24C	109.5
O3—C12—H12C	109.5	H24A—C24—H24C	109.5
H12A—C12—H12C	109.5	H24B—C24—H24C	109.5
H12B—C12—H12C	109.5	C11—O3—C12	116.21 (15)
C14—C13—H13A	109.5	C23—O6—C24	116.92 (16)

### Hydrogen-bond geometry (Å, °)

**Table:**

*D*—H···*A*	*D*—H	H···*A*	*D*···*A*	*D*—H···*A*
C24—H24A···O1^i^	0.96	2.53	3.467 (3)	164
C4—H4A···Cg1^ii^	0.93	3.17	3.858 (4)	133
C6—H6A···Cg2^iii^	0.93	3.26	4.051 (3)	144
C18—H18A···Cg1	0.93	3.20	3.940 (3)	138

Symmetry codes: (i) *x*, −*y*+1, *z*−1/2; (ii) *x*, −*y*, *z*−1/2; (iii) *x*, *y*−1, *z*.
